# Digital subtraction of temporally sequential mammograms for improved detection and classification of microcalcifications

**DOI:** 10.1186/s41747-021-00238-w

**Published:** 2021-09-14

**Authors:** Kosmia Loizidou, Galateia Skouroumouni, Costas Pitris, Christos Nikolaou

**Affiliations:** 1 KIOS Research and Innovation Center of Excellence, Department of Electrical and Computer Engineering, University of Cyprus, 1 Panepistimiou Avenue, Aglantzia, 2109 Nicosia, Cyprus; 2 Nicosia General Hospital, 215 Nicosia-Limassol Old Road, Strovolos, 2029 Nicosia, Cyprus; 3 Limassol General Hospital, 1 Nikaias, 4131 Limassol, Cyprus

**Keywords:** Breast cancer, Mammography, Radiographic image interpretation (computer-assisted), Retrospective studies, Machine learning

## Abstract

**Background:**

Our aim was to demonstrate that automated detection and classification of breast microcalcifications, according to Breast Imaging Reporting and Data System (BI-RADS) categorisation, can be improved with the subtraction of sequential mammograms as opposed to using the most recent image only.

**Methods:**

One hundred pairs of mammograms were retrospectively collected from two temporally sequential rounds. Fifty percent of the images included no (BI-RADS 1) or benign (BI-RADS 2) microcalcifications. The remaining exhibited suspicious findings (BI-RADS 4-5) in the recent image. Mammograms cannot be directly subtracted, due to tissue changes over time and breast deformation during mammography. To overcome this challenge, optimised preprocessing, image registration, and postprocessing procedures were developed. Machine learning techniques were employed to eliminate false positives (normal tissue misclassified as microcalcifications) and to classify the true microcalcifications as BI-RADS benign or suspicious. Ninety-six features were extracted and nine classifiers were evaluated with and without temporal subtraction. The performance was assessed by measuring sensitivity, specificity, accuracy, and area under the curve (AUC) at receiver operator characteristics analysis.

**Results:**

Using temporal subtraction, the contrast ratio improved ~ 57 times compared to the most recent mammograms, enhancing the detection of the radiologic changes. Classifying as BI-RADS benign *versus* suspicious microcalcifications, resulted in 90.3% accuracy and 0.87 AUC, compared to 82.7% and 0.81 using just the most recent mammogram (*p* = 0.003).

**Conclusion:**

Compared to using the most recent mammogram alone, temporal subtraction is more effective in the microcalcifications detection and classification and may play a role in automated diagnosis systems.

**Supplementary Information:**

The online version contains supplementary material available at 10.1186/s41747-021-00238-w.

## Key points

- The contrast ratio of the subtracted images was improved ~ 57 times, compared to that of the recent images without preprocessing.
- Eighteen per cent of pre-existing Breast Imaging Reporting and Data System (BI-RADS) category 2 findings were effectively removed and the remaining were detected with an accuracy of 94.1%.
- Accuracy and area under the curve of the classification of microcalcifications as BI-RADS 2 *versus* BI-RADS 4 or 5 were significantly higher with the use of temporal subtraction, compared to using only the most recent mammogram (*p* = 0.003).

## Background

Breast cancer screening with mammography is an effective approach to reduce breast cancer mortality. However, the large population involved and the use of double reading increases the workload and can limit the efficiency of the screening process [1]. To further exacerbate the challenge, various types of abnormalities are associated with breast cancer, including microcalcifications [2]. They can be benign or form microcalcification clusters possibly suspicious, to be managed appropriately. For the BI-RADS classification of microcalcifications as benign or suspicious, morphology, distribution, and change over time are key parameters [3]. Computer-aided diagnosis (CAD) systems are being explored as a means to improve the specificity of the classification of mammographic anomalies without compromising the sensitivity [4]. Several groups [5–7] have assessed the use of CAD systems for the detection of microcalcifications present in the most recent mammographic views, with sensitivity and specificity in the range of 82−89% and 87−88%, respectively, when discriminating between benign and suspicious microcalcifications. The main drawback of those systems is the considerable number of false positives (FPs) per image, that can range up to 1–3, which reduces their clinical applicability [8]. In addition, they provide no information regarding the presence of those abnormalities in previous mammographic sessions.

In temporal analysis, mammograms from multiple prior examinations are utilised. When prior information is available for direct comparison, abnormalities can be detected at an earlier stage and the radiologists feel more confident of their assessment [9]. Some studies have attempted to combine information from prior and recent mammographic views to reduce the FP and recall rates [9–14]. Prior and recent images were coarsely registered based on anatomical features (*e.g.,* nipple, skin, centre of mass which is the mean value across each dimension), and the locations of recently identified microcalcifications in the prior image were identified by regional registration, *i.e.,* searching around the location of the recent finding. Combining the features from both images resulted in an improved specificity, specificity, and reduced FPs rates. However, temporal analysis offers no benefit, over using just the recent mammographic view, when the findings are new with no traces of abnormality in the prior screening [11].

Going a step further, temporal subtraction begins with using both global and local features to register the entire breast areas of the recent and prior mammographic views. This allows direct subtraction of the images, digitally removing unchanged regions from the recent mammographic view, further delineating subtle recent changes, including microcalcifications [15, 16]. The objective of this study was to evaluate the effect of the subtraction of temporally sequential mammograms to eliminate unchanged features and improve the detection and classification of microcalcifications into benign and suspicious, based on their BI-RADS category. For comparison, the detection and classification methodologies were also applied to the most recent images without temporal subtraction.

## Methods

### Study population

**Fig. 1:**
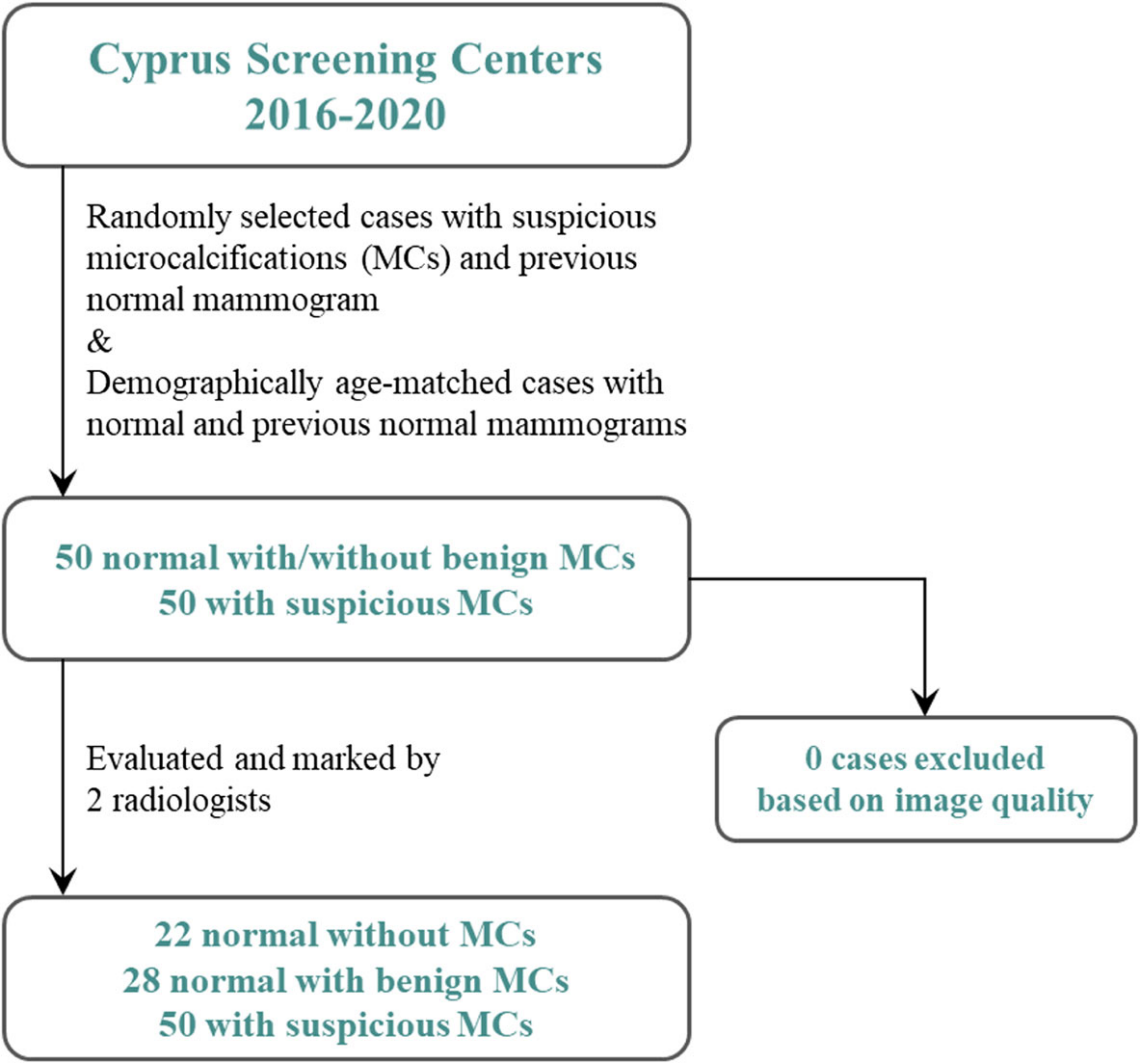
Flowchart of study population selection. *MCs* Microcalcifications

The current study expands on prior published work [16]. From the 100 participants that were eventually included, 80 were previously used for the purposes of describing the technical details of the algorithms employed. For this retrospective study, 100 pairs of full-field digital mammograms were collected, between 2012 and 2020, from various local hospitals (Nicosia General Hospital, Limassol General Hospital, Cyprus population screening program Aglantzia and Linopetra), performed by women (38 to 83 years of age, 60.07 ± 7.09, mean ± standard deviation) with either no microcalcifications or BI-RADS benign (normal population) or BI-RADS suspicious microcalcifications (suspicious population) in their current mammograms. A BI-RADS normal or benign prior mammogram (average interval of 2.2 years) was required for inclusion in the study (Fig. 1). The normal population was selected to form an age-matched group compared to the patients with BI-RADS suspicious microcalcifications. The study was approved by the appropriate Institutional Review Board (Cyprus National Bioethics Committee #EEBK ΕΠ 2020.01.144) and informed consent was retrospectively collected.

**Fig. 2:**
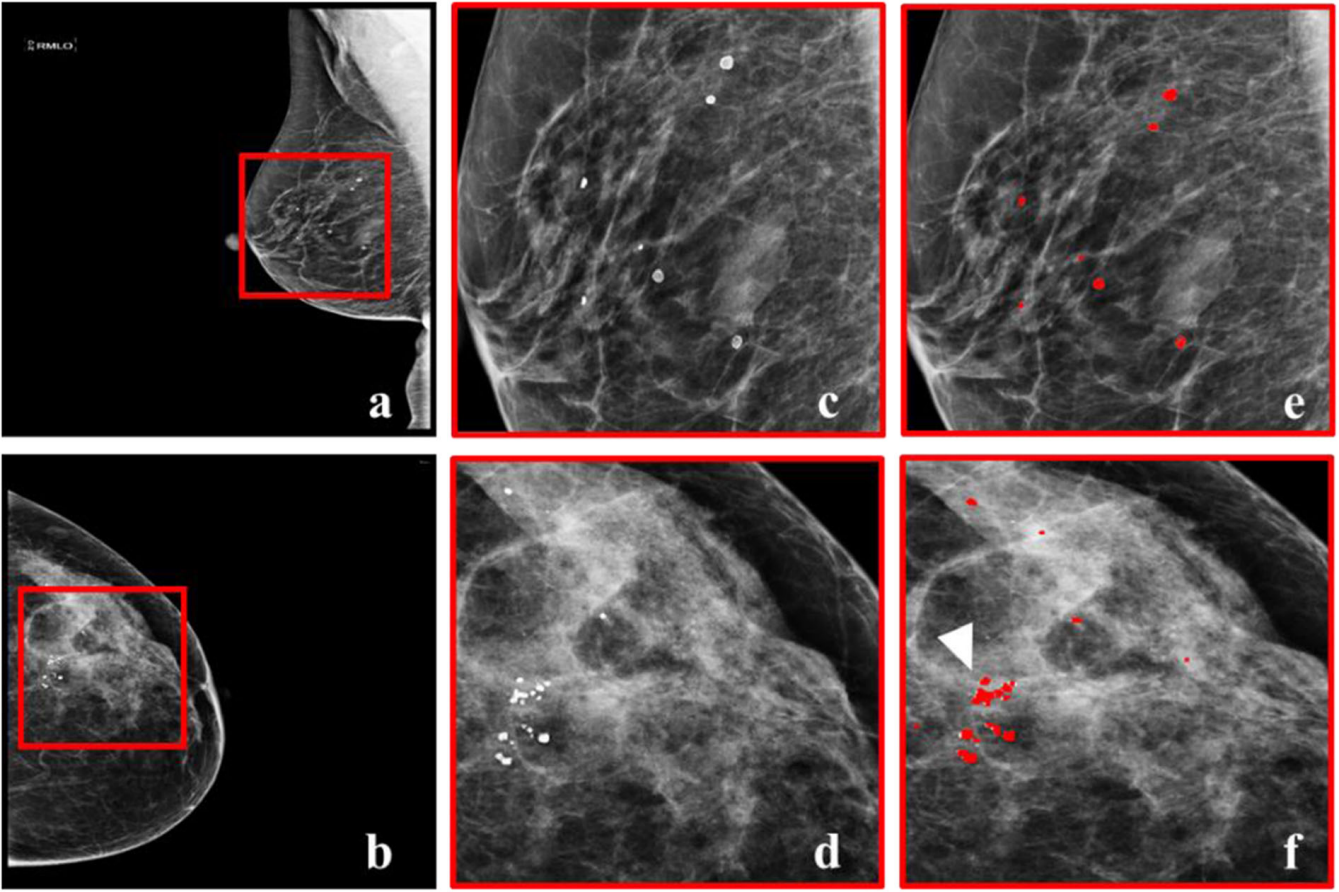
Dataset examples. **a** Most recent mammographic view of a woman (BI-RADS breast density class *b*) with BI-RADS benign microcalcifications. **b** Most recent mammographic view of woman (BI-RADS breast density class *c*) with BI-RADS benign and suspicious microcalcifications. **c**, **d** Zoomed regions from **a** and **b** showing microcalcifications. **e**, **f** The regions in **c** and **d** with precise marking of the location of microcalcifications, as annotated by two expert radiologists. The arrowhead in **f** points to a BI-RADS suspicious microcalcifications

For every participant, two mammographic views (cranio-caudal and medio-lateral oblique) of the breast showing the presence of micorcalcifications, from two sequential screening rounds, were included for a total of 400 images. A radiologist (C.N. with 10 years of experience) identified the patients to be included, according to the criteria specified above, and along with a second radiologist (G.S. with 2 years of experience), assessed the mammograms for assigning the BI-RADS category and marked the location of the microcalcifications. Interobserver agreement, *i.e.,* microcalcifications marked by both observers, was estimated at 97.1% for benign (BI-RADS 2) and 98.2% for suspicious (BI-RADS 4-5) microcalcifications; differences were resolved by consensus. A summary of the study population is shown in Table 1. Fifty percent of the mammograms came from healthy participants (28 with only BI-RADS benign microcalcifications in the prior and recent mammographic views and 22 with no visible microcalcifications). In the remaining 50%, at least one new BI-RADS suspicious microcalcification was present in the most recent mammographic view. The ground truth was based on the BI-RADS category, as evaluated by the radiologists, without any confirmation by follow-up (for “benign” lesions), or biopsy (for “suspicious” lesions). This data set not only included temporally sequential mammograms, but also precise annotation of each individual microcalcification to be used as a reference (Fig. 2). The data set included a total of 629 microcalcifications, 515 BI-RADS 2 and 114 BI-RADS 4 or 5. The size of the mammographic views was 4096 × 3328 pixels, in an 8-bit DICOM format. This data is publicly available (10.5281/zenodo.5036062).

**Table 1:** Characteristics of the population and digital mammography examinations selected for the study

Variable	Population		
	BI-RADS normal (***n*** = 50)	BI-RADS suspicious (***n*** = 50)	Total (***n*** = 100)
**Patient age**			
Mean ± standard deviation	59.42 ± 5.97	60.72 ± 8.00	60.07 ± 7.09
Median	59	61.5	59.5
Range	47−75	38−83	38−83
Interquartile range	55−64	55.75−66.25	55−65
**BI-RADS breast density**			
*a*	5	5	10
*b*	33	24	57
*c*	10	18	28
*d*	2	3	5
**BI-RADS classification**			
1	22	0	22
2	28	0	28
3	0	0	0
4a	0	27	27
4b	0	15	15
4c	0	6	6
5	0	2	2

### Image registration, subtraction, and segmentation

Supplemental Fig. S1 shows the diagram of the proposed methodology for detection and BI-RADS classification of microcalcifications. The procedure began with image preprocessing for normalisation, border removal [17], and gamma correction [18]. Next, each prior mammographic view was co-registered to the most recent one. Image registration is a critical step in temporal subtraction, since it corrects for the changes that occur in the breast over time and due to deformation between mammograms. Demons registration [19], a non-rigid method based on local flows, was employed due to superior performance compared to other common approaches [20]. The prior registered mammographic view was subsequently subtracted from the recent, effectively removing the regions that have remained unchanged since the previous exam. The contrast ratio of the subtracted image, *i.e.,* the ratio of the maximum divided by the average intensity, was compared to that of the recent mammographic view to evaluate the effectiveness of the removal of the background. Furthermore, the capability of the subtraction to remove unchanged microcalcifications, thus reducing the FP rate, was also assessed. After range filtering [21], the intensity values of each image were converted to binary, *i.e.,* 0 or 1, using an intensity threshold, obtained by optimising the global BI-RADS classification rate. The binary image was further processed morphologically. The operations of closing (removing small unconnected regions) and opening (filling small gaps) were applied and the remaining regions were considered as possible microcalcifications.

### Feature extraction and selection for classification

To differentiate the true microcalcifications from other tissue and, subsequently, the BI-RADS benign from the BI-RADS suspicious microcalcifications, using machine learning algorithms, several features were estimated from every possible region containing microcalcifications. Ninety-six, shape, intensity first order statistic, and grey-level co-occurrence matrix (GLCM) features were extracted [22–24]. The GLCM was calculated at 0﻿°, 45﻿°, 90﻿°, and 135° and 5-, 15-, and 25-pixel offset. The mean and standard deviation of each GLCM texture property were obtained, resulting in 72 features. Hypothesis test (*t-*test) [25] and feature importance [26] were employed to identify the most significant features and further evaluate their contribution to the classification.

### Training and comparison of classifier designs

For the BI-RADS classification, 9 classifiers were evaluated: 9-nearest neighbors [1], decision trees [27], random forest [27], multi-layer perceptron [28], adaptive boosting [29], bagging [30], gradient boosting [31], and ensemble voting [32]. In addition, different neural network configurations were evaluated using Python (Python Software Foundation, Wilmington, USA, v. 3.7.7) and Keras (Keras Special Interest Group, François Chollet, Mountain View, USA, v. 2.3.1) [33]. The resulting, most suitable, network consisted of 7 fully connected layers, with 986,738 trainable parameters. Rectified linear unit was used as an activation function and adaptive dropout regularisation was included every two hidden layers. Gaussian noise was added after dropout, as a regularisation term, in order to increase the robustness of the network. Batch size was set to 128 and the network was trained for 100 epochs. Traditional classifiers were selected based on their prior application to mammography.

The complete dataset was used during the training stage with leave-one-patient-out (LOPO) cross-validation. This cross-validation approach was critical in order to avoid bias from including images of the same patient in both test and training sets. K-fold cross-validation was also considered, again by dividing the patients into folds. Initially, the possible microcalcifications were classified as normal tissue or true microcalcifications and, subsequently, the true microcalcifications were classified as BI-RADS benign or suspicious.

### Detection and classification using the most recent mammogram alone

For comparison purposes, the same classification approach was optimised and applied to the most recent mammograms, without temporal subtraction, to verify the benefit of temporal subtraction.

### Statistical analysis

The classification performance was evaluated by computing the sensitivity, specificity, accuracy, and the area under the curve (AUC) at receiver operator characteristics analysis. The cut-off values for calculating sensitivity and specificity were selected optimally so that BI-RADS false positive and false negative numbers are minimised, *i.e.,* the cross point of the positive and negative distributions. In the comparison of the results from using just the most recent mammogram, the Fisher test was used with a level of statistical significance set to *p* = 0.05.

## Results

### Image registration, subtraction, and segmentation

**Fig. 3:**
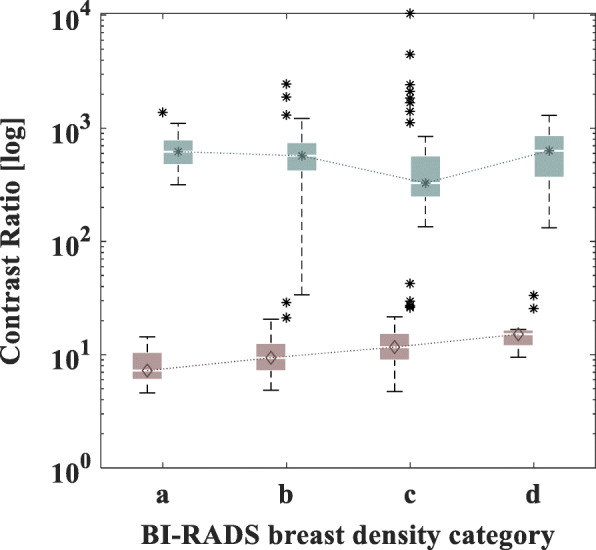
Plot comparing the contrast ratio, in logarithmic scale, of the unprocessed recent image and the image created by temporal subtraction, for the four categories of BI-RADS breast density. The contrast ratio increased indicating that temporal subtraction is successful for all breast densities

**Fig. 4:**
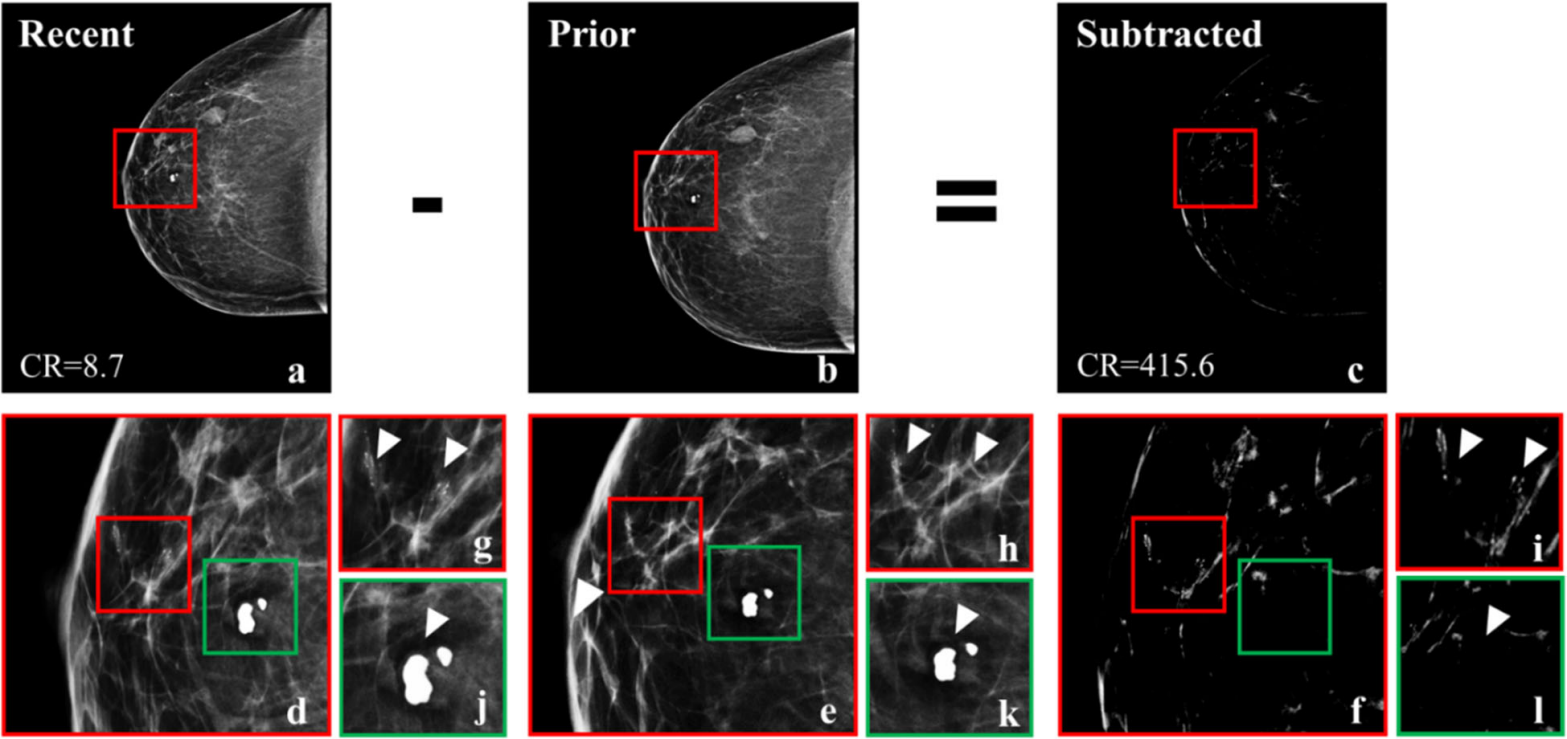
Example of temporal subtraction in a woman (BI-RADS breast density class *b*) with BI-RADS benign and suspicious microcalcifications. **a** Most recent mammographic view. **b** Prior mammographic view. **c** The result of subtracting the registered version of **b** from **a**, where the contrast ratio has increased 92 times after subtraction. **d**−**f** Zoomed regions marked by the red squares in **a**−**c**. **g**–**i** Zoomed regions marked by the red squares in **d**−**f** where the arrowheads point to new BI-RADS suspicious microcalcifications, which were not subtracted. (**j**−**l**) Zoomed regions marked by the green squares in **d**−**f** where the arrow points to pre-existing BI-RADS benign microcalcifications, which were completely subtracted. *CR* Contrast ratio

Image registration and subtraction yielded an average 72% reduction of image intensity, a result of removing structures that have remained unchanged between screenings. The average contrast ratio of the subtracted images was ~ 57 times higher compared to the recent mammographic view (Fig. 3). Eighteen percent of old BI-RADS benign microcalcifications were effectively removed (Table 2). It is also important to note that none of the BI-RADS suspicious microcalcifications were removed by this process (Fig. 4). The processing time for these operations was an average of ~ 15 min per image pair (Intel® Core™ i7 2 GHz; Intel Corp., Santa Clara, CA, USA).

**Table 2:** Elimination of old microcalcifications that appear in both screening rounds, in BI-RADS normal and suspicious mammograms

Mammograms	New microcalcifications	Old microcalcifications	Overlapping microcalcifications	Overlap (%)		Reduction (%)	
**BI-RADS normal** **(*n =*200)**	248	224	54	54/224	(24.1)	54/248	(21.8)
**BI-RADS suspicious** **(*n =*200)**	398	319	64	64/319	(20.1)	64/398	(16.1)
**Total** **(*n =*400)**	646	543	118	118/543	(21.7)	118/646	(18.3)

### Feature extraction and selection for classification

Features of microcalcifications were extracted from the images as described in the previous section. Based on the results of *t-*test and feature importance, the features with the most significant contribution to classification were identified for each classification round. The details of the features that have been selected in each case can be found in Supplemental Table S1.

### Detection and classification using temporal subtraction

**Fig. 5:**
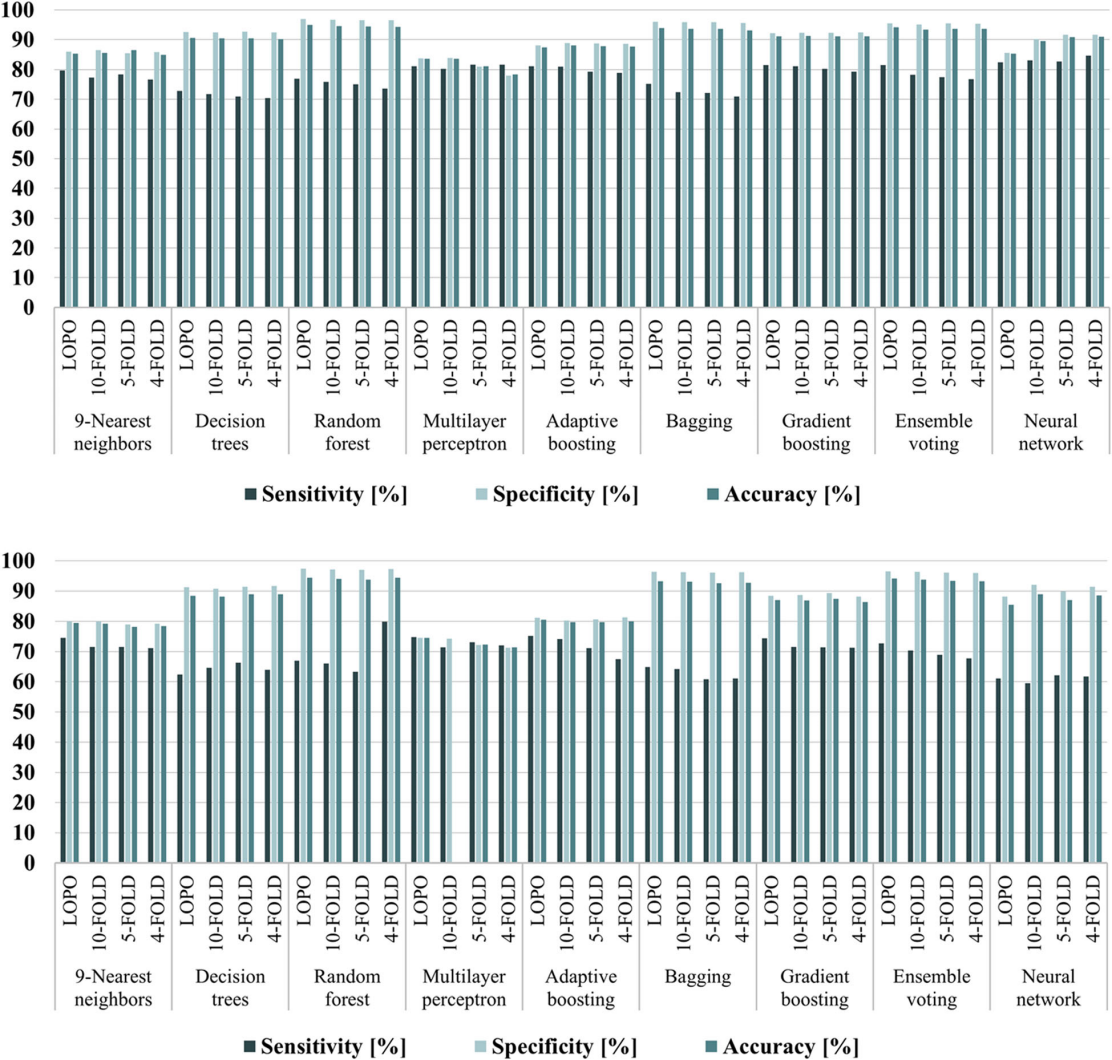
Classification results of the possible microcalcifications as radiologically normal tissue or true microcalcifications using different classifiers and cross-validation methods. **up** Results using temporal subtraction of mammograms. **down** Results using only the most recent mammograms. *LOPO* Leave-one-patient-out

The results of the detection of the microcalcifications are summarised in Table 3. The sensitivity, specificity, accuracy, and AUC of the different methods were in the range of 72.8−82.3%, 83.7−97%, 83.5−95%, and 0.82−0.88%, respectively. Based on the AUC, the most successful classification scheme was the ensemble voting with 81.4% sensitivity, 95.5% specificity, 94.1% accuracy, and 0.88 AUC. The application of k-fold cross-validation (Fig. 5a), using *k* = 4, 5, and 10, verified that the performance remained approximately at the same level.

**Table 3:** Comparison of the classification results of the possible microcalcifications as normal tissue or radiologically true microcalcifications with temporal subtraction (TS) of mammograms or using only the most recent mammograms (RM), in a leave-one-patient-out cross-validation scheme

Classifier	Sensitivity (%)			Specificity (%)			Accuracy (%)			AUC	
**9-Nearest** **neighbors**	TS RM	501/629 469/629	(79.7) (74.6)	TS RM	4928/5739 4593/5739	(85.9) (80.0)	TS RM	5429/6368 5062/6368	(85.3) (79.5)	TS RM	0.83 0.76
**Decision** **trees**	TS RM	458/629 392/629	(72.8) (62.3)	TS RM	5311/5739 5234/5739	(92.5) (91.2)	TS RM	5769/6368 5626/6368	(90.6) (88.4)	TS RM	0.83 0.78
**Random** **forest**	TS RM	484/629 421/629	(77.0) (66.9)	TS RM	5565/5739 5591/5739	(97.0) (97.4)	TS RM	6049/6368 6012/6368	(95.0) (94.4)	TS RM	0.87 0.82
**Multilayer** **perceptron**	TS RM	510/629 470/629	(81.1) (74.7)	TS RM	4806/5739 4275/5739	(83.7) (74.5)	TS RM	5316/6368 4745/6368	(83.5) (74.5)	TS RM	0.82 0.73
**Adaptive** **boosting**	TS RM	510/629 473/629	(81.1) (75.2)	TS RM	5561/5739 4659/5739	(88.0) (81.2)	TS RM	6071/6368 5132/6368	(87.3) (80.6)	TS RM	0.85 0.77
**Bagging**	TS RM	473/629 408/629	(75.2) (64.9)	TS RM	5510/5739 5528/5739	(96.0) (96.3)	TS RM	5983/6368 5936/6368	(94.0) (93.2)	TS RM	0.86 0.80
**Gradient** **boosting**	TS RM	512/629 468/629	(81.4) (74.4)	TS RM	5291/5739 5073/5739	(92.2) (88.4)	TS RM	5803/6368 5541/6368	(91.1) (87.0)	TS RM	0.87 0.80
**Ensemble** **voting**	**TS** **RM**	**512/629** **457/629**	**(81.4)** **(72.8)**	**TS** **RM**	**5480/5739** **5536/5739**	**(95.5)** **(96.5)**	**TS** **RM**	**5992/6368** **5993/6368**	**(94.1)** **(94.1)**	**TS** **RM**	**0.88** **0.83**
**Neural** **network**	TS RM	518/629 384/629	(82.4) (61.1)	TS RM	4911/5739 5058/5739	(85.6) (88.1)	TS﻿﻿ RM	5429/6368 5448/6368	(85.3) (85.5)	TS RM	0.84 0.76

*AUC* Area under the curve

**Fig. 6:**
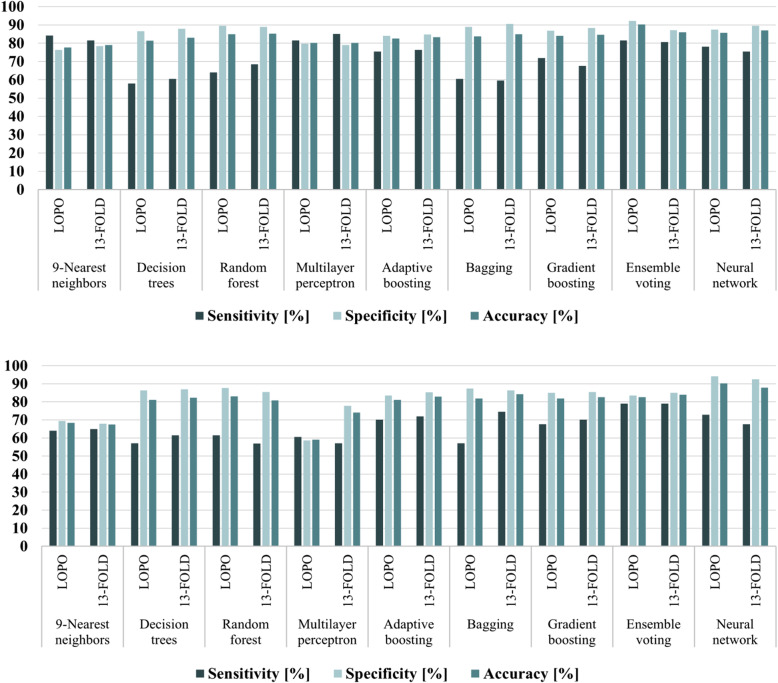
Classification results of the true microcalcifications as BI-RADS benign or suspicious using different classifiers and cross-validation methods. **up** Results using temporal subtraction of mammograms. **down** Results using only the most recent mammograms. *LOPO* Leave-one-patient-out

The optimisation of the various classifiers for the classification of microcalcifications as benign or suspicious according to their BI-RADS category, using LOPO cross-validation, resulted in the outcomes summarised in Table 4. The sensitivity, specificity, accuracy and AUC were in the range of 57.9−84.2%, 77.8−92.2%, 79.0−90.3% and, 0.70−0.87%, respectively. Based on the AUC, the most successful classification scheme was again ensemble voting with 81.6% sensitivity, 92.2% specificity, 90.3% accuracy, and 0.87 AUC. For this classification round, only 13-fold cross-validation was applied (Fig. 6a) due to the smaller number of patients with microcalcifications (*i.e.,* 78 patients with true microcalcifications, 50 BI-RADS suspicious, and 28 BI-RADS normal with benign microcalcifications). Example images visually demonstrating the classification outcome, using temporal subtraction, where BI-RADS benign and suspicious microcalcifications were correctly identified, are shown in Supplemental Fig. S2.

**Table 4:** Comparison of the classification results of the true microcalcifications as BI-RADS benign or suspicious using temporal subtraction (TS) of mammograms and using only the most recent mammograms (RM), in a leave-one-patient-out cross-validation scheme

Classifier	Sensitivity (%)			Specificity (%)			Accuracy (%)			AUC	
**9-Nearest** **neighbors**	TS RM	96/114 73/114	(84.2) (64.0)	TS RM	393/515 357/515	(76.3) (69.3)	TS RM	489/629 430/629	(77.7) (68.4)	TS RM	0.80 0.67
**Decision** **trees**	TS RM	66/114 65/114	(57.9) (57.0)	TS RM	446/515 445/515	(86.6) (86.4)	TS RM	512/629 510/629	(81.4) (81.1)	TS RM	0.72 0.72
**Random** **forest**	TS RM	73/114 70/114	(64.0) (61.4)	TS RM	461/515 452/515	(89.5) (87.8)	TS RM	534/629 522/629	(84.9) (83.0)	TS RM	0.77 0.75
**Multilayer** **perceptron**	TS RM	93/114 69/114	(81.6) (60.5)	TS RM	411/515 302/515	(79.8) (58.6)	TS RM	504/629 371/629	(80.1) (59.0)	TS RM	0.81 0.6
**Adaptive** **boosting**	TS RM	86/114 80/114	(75.4) (70.2)	TS RM	433/515 430/515	(84.1) (83.5)	TS RM	519/629 510/629	(82.5) (81.1)	TS RM	0.8 0.77
**Bagging**	TS RM	69/114 65/114	(60.5) (57.0)	TS RM	458/515 450/515	(88.9) (87.4)	TS RM	527/629 515/629	(83.8) (81.9)	TS RM	0.75 0.72
**Gradient** **boosting**	TS RM	82/114 77/114	(71.9) (67.5)	TS RM	447/515 438/515	(86.8) (85.1)	TS RM	529/629 515/629	(84.1) (81.9)	TS RM	0.79 0.76
**Ensemble** **voting**	**TS** **RM**	**93/114** **90/114**	**(81.6)** **(79.0)**	**TS** **RM**	**475/515** **430/515**	**(92.2)** **(83.5)**	**TS** **RM**	**568/629** **520/629**	**(90.3)** **(82.7)**	**TS** **RM**	**0.87** **0.81**
**Neural** **network**	TS RM	89/114 83/114	(78.1) (72.8)	TS RM	450/515 485/515	(87.4) (94.2)	TS﻿ RM	539/629 568/629	(85.7) (90.3)	TS RM	0.83 0.83

*AUC* Area under the curve

### Detection and classification using the most recent mammogram alone

Table 3 shows the classification results for the identification of true microcalcifications using features selected only from the most recent mammographic view (Supplemental Table S1) and the same classifiers as before optimised for these features. The best classification performance was achieved using ensemble voting with 72.7% sensitivity, 96.5% specificity, 94.1% accuracy, and 0.83 AUC. Subsequently, the true microcalcifications were classified as BI-RADS benign or suspicious and the results are presented in Table 4. Again, the ensemble voting was the most successful method, providing 78.9% sensitivity, 83.5% specificity, 82.7% accuracy, and 0.81 AUC. As before, k-fold cross-validation was also performed, using the same values of *k* (Figs. 5b and 6b) showing that the algorithm was also stable and robust.

## Discussion

A method for the detection and classification of microcalcifications, according to their BI-RADS category, from the subtraction of temporally sequential mammographic views was developed. The aim of this work was to combine temporal subtraction with machine learning in order to enhance the contrast ratio, eliminate the radiologically unchanged microcalcifications and, most importantly, improve the classification accuracy of microcalcifications as benign or suspicious, based on their BI-RADS categories. For effective and efficient subtraction of the prior from the most recent mammographic view, preprocessing, registration, and postprocessing procedures were applied. Machine learning techniques were then used to eliminate the FPs, *i.e.,* normal tissue misclassified as microcalcifications, and, furthermore, to classify the true microcalcifications as BI-RADS benign or suspicious. Bagging, gradient boosting, ensemble voting, and neural networks are more recent additions to machine learning and were chosen for their potential to provide improved classification. Ideally, the proposed method should have also been verified on an independent external dataset. However, publicly available datasets do not provide sequential mammograms or images annotated at the level of individual microcalcifications.

Demons registration [19] was very effective in matching the prior to the recent mammographic views, since it accounted for the complex transformations and distortions that appear between screenings. Using the proposed technique, the contrast ratio improved ~ 57 times enhancing the contrast of the recent changes in the images. The elimination of most of the background and unchanged BI-RADS benign microcalcifications can make the radiologic evaluation of mammograms and the detection of even subtle abnormalities, easier and faster. This reduces the effort and time expended by the radiologist by enhancing the new and, most probably, more diagnostically useful information.

With automated BI-RADS classification, the proposed method achieved 81.4% sensitivity, 95.5% specificity and 94.1% accuracy for the detection of true microcalcifications and 81.6% sensitivity, 92.2% specificity, and 90.3% accuracy for the classification of microcalcifications as BI-RADS benign or suspicious, both using an optimised feature set and an ensemble voting model. This high accuracy demonstrates the effectiveness of the algorithm, which provided statistically superior performance over using only the most recent mammograms. The average classification accuracy for the characterisation of true microcalcifications as BI-RADS benign or suspicious improved by 7% with the introduction of temporal subtraction (*p* = 0.003). To evaluate the robustness of the method, k-fold cross-validation was implemented, in addition to LOPO cross-validation. The algorithm performed at approximately the same level in all the cross-validation scenarios, indicating that the proposed method is robust and should be able to function equally well as new data becomes available.

The main limitation of this study is the relatively limited dataset acquired from local hospitals with a single protocol. Even though the results presented here are promising, more sequential pairs are required to definitively prove the generalizability of the proposed algorithm. Unfortunately, publicly available databases cannot be exploited for the purposes of this project, since they neither contain sequential mammograms nor they include images annotated at the level of individual microcalcifications as in this study. Another limitation is the fact that although the suspicious microcalcifications were identified by two expert radiologists, the BI-RADS classification of clusters of microcalcifications not only varies from one radiologist to another, but might also be disproved by follow-up or pathology. In addition, the adoption of the BI-RADS classification as the ground truth, without any confirmation by follow-up or pathology, limits the generalizability and the ultimate utility of the tool. This is a consequence of mimicking the human reader rather than offering an unbiased opinion based on the true and confirmed state of the microcalcifications.

The results of this study cannot be readily and directly compared to other state-of-the-art techniques described in the literature for various reasons. The existing and freely available image databases contain only one mammogram per patient (*i.e.,* no prior information). In addition, in several cases, entire images are classified rather than individual microcalcifications [34]. Furthermore, in most state-of-the-art algorithms, cross-validation is implemented by randomly dividing the microcalcifications into training and test sets, or by using part of the same image in the test and another part in the training set [35]. Such approaches can introduce bias, which results in artificially improved classification results. In general, most studies in the literature report accuracy and AUC, which in the case of benign *versus* malignant classification of microcalcifications range between 80−89% and 0.86−0.92% (Supplemental Table S2). In this study, a more appropriate approach was selected, which assigned the entire data set corresponding to a patient either to the training or the test set, and performed LOPO cross-validation. In order to prove the effectiveness of temporal subtraction in a fair manner, the results were compared to an optimised algorithm using only the most recent mammograms of the same dataset.

There are no examples of temporal analysis for microcalcification identification and classification in the literature. Furthermore, there are only a few studies on the use of temporal analysis for the characterisation of mass lesions [9–11, 13]. Rather than image subtraction, their approach was to extract several features from the recent and prior mammograms separately and, then, combine them to improve the classification accuracy. Their results confirmed that the use of prior information could improve the detection and classification of mass lesions. However, temporal analysis offers no benefit when there is no abnormality in the prior screening.

In conclusion, the proposed technique demonstrates that temporal subtraction achieves superior performance in the detection and classification of microcalcifications, based on their BI-RADS category, compared to using only the most recent mammograms. The inclusion of more patients as well as the extension of the approach to detect and classify other abnormalities in mammograms (*e.g.,* breast masses or distortions) can further enhance the diagnostic potential of temporal subtraction. In the future, the proposed methodology has the potential to substantially contribute to the development of automated CAD systems to assist in the radiologic classification of breast abnormalities and serve as a “second reader” or a “tie breaker” especially in low resource settings.

## Supplementary Information

**Additional file1 **Supplemental Fig. S1 Diagram of the proposed methodology for the detection and BI-RADS classification of breast microcalcifications using temporal subtraction of sequential mammograms. Supplemental Table S1 Features selected for the 1st and 2nd round of BI-RADS classification using temporal subtraction and only the most recent mammograms. Supplemental Fig. S2 Results of the classification of the radiologically true microcalcifications as BI-RADS benign or suspicious in the most recent mammographic view of a woman (BI-RADS breast density class *b*). (a) Most recent mammographic view, with green circles around the BI-RADS benign microcalcifications, and a red circle around the BI-RADS suspicious microcalcifications. (b) Zoomed view of the red square in a with BI-RADS suspicious microcalcifications. (c) Zoomed view of the green square in a with BI-RADS benign microcalcifications. Supplemental Table S2 Comparison of accuracy and AUC of different state-of-the-art techniques for the classification of benign *versus* malignant microcalcifications [36–40].

## Data Availability

The dataset generated during the current study is publicly available through Zenodo. The dataset can be accessed using the following link: 10.5281/zenodo.5036062.

## Abbreviations

AUC: Area under the curve
BI-RADS: Breast Imaging Reporting and Data System
CAD: Computer-aided diagnosis
FP: False positive
GLCM: Grey-level co-occurrence matrix
LOPO: Leave-one-patient-out
