# Supplementary material for: Digital subtraction of temporally sequential mammograms for improved detection and classification of microcalcifications
Source: Eur Radiol Exp. 2021 Sep 14;5:40. doi: 10.1186/s41747-021-00238-w (PMC8440760; doi:10.1186/s41747-021-00238-w)
Supplement: Supplementary file 1 — Additional file1 Supplemental Fig. S1 Diagram of the proposed methodology for the detection and BI-RADS classification of breast microcalcifications using temporal subtraction of sequential mammograms. Supplemental Table S1 Features selected for the 1st and 2nd round of BI-RADS classification using temporal subtraction and only the most recent mammograms. Supplemental Fig. S2 Results of the classification of the radiologically true microcalcifications as BI-RADS benign or suspicious in the most recent mammographic view of a woman (BI-RADS breast density class b). (a) Most recent mammographic view, with green circles around the BI-RADS benign microcalcifications, and a red circle around the BI-RADS suspicious microcalcifications. (b) Zoomed view of the red square in a with BI-RADS suspicious microcalcifications. (c) Zoomed view of the green square in a with BI-RADS benign microcalcifications. Supplemental Table S2 Comparison of accuracy and AUC of different state-of-the-art techniques for the classification of benign versus malignant microcalcifications [36–40]. [file 41747_2021_238_MOESM1_ESM.docx]

**ELECTRONIC SUPPLEMENTARY MATERIAL**

| 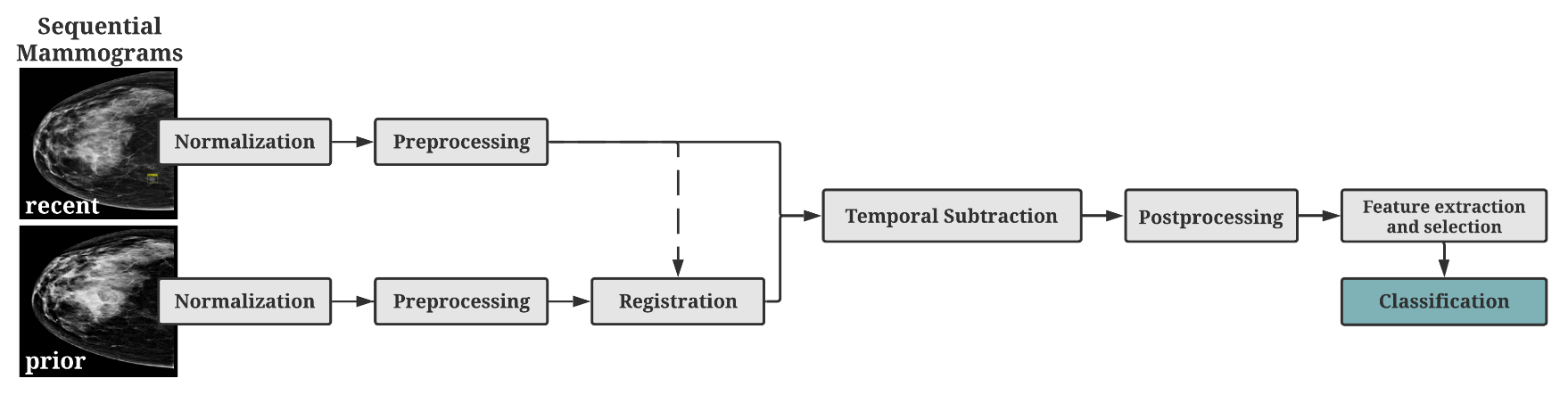 |
| --- |
| **Supplemental Fig. S1** Diagram of the proposed methodology for the detection and BI-RADS classification of breast microcalcifications using temporal subtraction of sequential mammograms. |

| **Supplemental Table S1** Features selected for the 1^st^ and 2^nd^ round of BI-RADS classification using temporal subtraction and only the most recent mammograms | | | |
| --- | --- | --- | --- |
| **Temporal subtraction** | | **Recent mammogram** | |
| **1^st^ round** | **2^nd^ round** | **1^st^ round** | **2^nd^ round** |
| Area | Area | Convex area | Euler number |
| Convex area | Eccentricity | Euler number | Filled area |
| Eccentricity | Equivalent diameter | Extent | Min intensity |
| Equivalent diameter | Major axis length | Min intensity | Orientation |
| Extent | Minor axis length | Orientation | Average intensity |
| Filled area | Perimeter | Perimeter | Skewness |
| Major axis length | Solidity | Average intensity | Contrast 135° D1 |
| Minor axis length | Entropy | Kurtosis | Contrast mean D1 |
| Perimeter | Skewness | Skewness | Contrast STD D1 |
| Minimum intensity | Energy 0° D1 | Standard deviation | Correlation 0° D1 |
| Entropy | Energy 45° D1 | Variance | Correlation STD D1 |
| Kurtosis | Energy 90° D1 | Contrast 90° D1 | Energy 45° D1 |
| Skewness | Energy mean D1 | Contrast 135° D1 | Homogeneity 45° D1 |
| Standard deviation | Homogeneity 0° D1 | Contrast mean D1 | Homogeneity STD D1 |
| Contrast 0° D1 | Homogeneity 45° D1 | Correlation 0° D1 | Contrast 0° D2 |
| Contrast 45° D1 | Homogeneity 90° D1 | Energy STD D1 | Contrast 45° D2 |
| Contrast 90° D1 | Homogeneity 135° D1 | Homogeneity STD D1 | Contrast 90° D2 |
| Contrast 135° D1 | Homogeneity mean D1 | Contrast 45° D2 | Contrast 135° D2 |
| Contrast mean D1 | Correlation 90° D2 | Contrast 90° D2 | Correlation 45° D2 |
| Correlation mean D1 | Energy 45° D2 | Contrast mean D2 | Correlation mean D2 |
| Contrast 0° D2 | Energy mean D2 | Correlation 0° D2 | Correlation STD D2 |
| Contrast 45° D2 | Homogeneity 0° D2 | Homogeneity 0° D2 | Energy 135° D2 |
| Contrast 90° D2 | Homogeneity 45° D2 | Contrast 45° D3 | Homogeneity STD D2 |
| Contrast mean D2 | Homogeneity 90° D2 | Contrast 90° D3 | Contrast 45° D3 |
| Contrast 0° D3 | Homogeneity 135° D2 | Contrast 135° D3 | Contrast mean D3 |
| Contrast 45° D3 | Homogeneity mean D2 | Correlation mean D3 | Contrast STD D3 |
| Contrast 90° D3 | Correlation 45° D3 | Correlation STD D3 | Energy 45° D3 |
| Contrast 135° D3 | Homogeneity 0° D3 | Energy 0° D3 | Energy mean D3 |
| Contrast mean D3 | Homogeneity 135° D3 | Shape ratio | Homogeneity STD D3 |
| Contrast STD D3 | Homogeneity mean D3 | Smoothness | Compactness |
| *STD* Standard deviation |  |  |  |

| 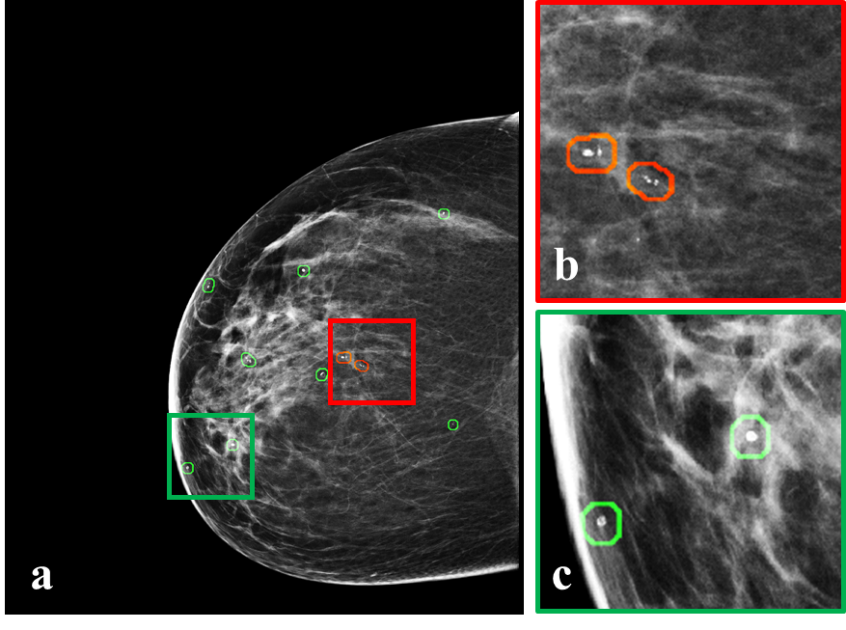 |
| --- |
| **Supplemental Fig. S2** Results of the classification of the radiologically true microcalcifications as BI-RADS benign or suspicious in the most recent mammographic view of a woman (BI-RADS breast density class *b*). **(a)** Most recent mammographic view, with green circles around the BI-RADS benign microcalcifications, and a red circle around the BI-RADS suspicious microcalcifications. **(b)** Zoomed view of the red square in **a** with BI-RADS suspicious microcalcifications. **(c)** Zoomed view of the green square in **a** with BI-RADS benign microcalcifications. |

| **Supplemental Table S2** Comparison of accuracy and area under the curve (AUC) of different state-of-the-art techniques for the classification of benign versus malignant microcalcifications | | | | | |
| --- | --- | --- | --- | --- | --- |
| **Method** | **Number of regions of interest** | **Feature**  **type** | **Classifier** | **Accuracy**  **(%)** | **AUC** |
| Ren et al. (2012) [36] | 295 | Statistical features | k-Nearest  neighbors | 82 | 0.86 |
| Khehra et al. (2013) [37] | 380 | Statistical, shape, and textural features | Least-Square Support Vector Machine | 89 | 0.89 |
| Strange et al. (2014) [38] | 300 | Mereotopological features | Barcodes | 80 | 0.82 |
| Chen et al. (2015) [39] | 300 | Topological features | k-Nearest  neighbors | 85 | 0.91 |
| Fanizzi et al. (2020) [40] | 260 | Textural features and interest points/corners | Random  forest | 88 | 0.92 |
| AUC Area under the curve | | | | | |
